# Clinical outcomes and risk factors for mortality in recipients with carbapenem-resistant gram-negative bacilli infections after kidney transplantation treated with ceftazidime-avibactam: a retrospective study

**DOI:** 10.3389/fcimb.2024.1404404

**Published:** 2024-05-08

**Authors:** Fei Zhang, Pengfei Li, Jinbiao Zhong, Handong Ding, Guiyi Liao, Chaozhao Liang

**Affiliations:** ^1^ Department of Urology, The First Affiliated Hospital of Anhui Medical University, Hefei, Anhui, China; ^2^ Institute of Urology, The First Affiliated Hospital of Anhui Medical University, Hefei, Anhui, China; ^3^ Anhui Province Key Laboratory of Urological and Andrological Diseases Research and Medical Transformation, Anhui Medical University, Hefei, Anhui, China

**Keywords:** Carbapenem-resistant gram-negative bacilli, ceftazidime-avibactam, kidney transplant, infections, carbapenem-resistant *Klebsiella pneumoniae*

## Abstract

**Background:**

Ceftazidime-avibactam is a treatment option for carbapenem-resistant gram-negative bacilli (CR-GNB) infections. However, the risk factors associated with ceftazidime-avibactam (CAZ-AVI) treatment failure in kidney transplant (KT) recipients and the need for CAZ-AVI-based combination therapy remain unclear.

**Methods:**

From June 2019 to December 2023, a retrospective observational study of KT recipients with CR-GNB infection treated with CAZ-AVI was conducted, with the primary outcome being 30-day mortality and secondary outcomes being clinical cure, microbiological cure, and safety. Risk factors for 30-day mortality and clinical failure were also investigated.

**Results:**

A total of 81 KT recipients treated with CAZ-AVI were included in this study. Forty recipients (49.4%) received CAZ-AVI monotherapy, with a 30-day mortality of 22.2%. The clinical cure and microbiological cure rates of CAZ/AVI therapy were 72.8% and 66.7%, respectively. CAZ-AVI alone or in combination with other medications had no effect on clinical cure or 30-day mortality. Multivariate logistic regression analysis revealed that a higher Acute Physiology and Chronic Health Evaluation (APACHE) II score (odds ratio [OR]: 4.517; 95% confidence interval [CI]: 1.397-14.607; *P* = 0.012) was an independent risk factor for 30-day mortality. Clinical cure was positively associated with the administration of CAZ-AVI within 48 hours of infection onset (OR: 11.009; 95% CI: 1.344-90.197; *P*=0.025) and negatively associated with higher APACHE II scores (OR: 0.700; 95% CI: 0.555-0.882; *P*=0.002). Four (4.9%) recipients experienced recurrence within 90 days after the initial infection, 3 (3.7%) recipients experienced CAZ-AVI-related adverse events, and no CAZ-AVI resistance was identified.

**Conclusion:**

CAZ-AVI is an effective medication for treating CR-GNB infections following kidney transplantation, even as monotherapy. Optimization of CAZ/AVI therapy (used within 48 hours of infection onset) is positively associated with potential clinical benefit. Further larger-scale studies are needed to validate these findings.

## Introduction

With the widespread use of carbapenem antibiotics in recent years, the isolation rate of carbapenem-resistant gram-negative bacilli (CR-GNB), primarily carbapenem-resistant Enterobacteriaceae (CRE), carbapenem-resistant *Pseudomonas aeruginosa* (CRPA) and carbapenem-resistant *Acinetobacter baumannii* (CRAB), has increased (Doi, 2019). Kidney transplant (KT) recipients are particularly vulnerable to CR-GNB infection due to factors such as severe surgical trauma and compromised immune function. These infections are often associated with high clinical failure, morbidity, and mortality due to limited treatment options (Bodro et al., 2015; Zhang et al., 2021). In China, the main antibiotics used to treat infections caused by these refractory isolates are aminoglycosides, polymyxin and tigecycline. However, concerns about their clinical efficacy, safety, and emerging resistance have limited their clinical use (Nang et al., 2021; Yahav et al., 2021). Especially most of the above drugs are nephrotoxic, and considering the protection of transplanted kidney function, the use of these drugs is further limited in KT recipients. Clinically, the treatment options for CR-GNB infection following kidney transplantation are minimal, and new therapeutic approaches against these refractory pathogens are urgently needed.

Ceftazidime-avibactam (CAZ-AVI), a novel β-lactam/β-lactamase inhibitor, was approved by the China National Medical Products Administration on May 21, 2019, for the treatment of complicated abdominal and urinary tract infections, hospital-acquired, ventilator-associated pneumonia, and infections caused by aerobic gram-negative bacteria in adult patients with limited treatment options (van Duin and Bonomo, 2016; Torres et al., 2018). Avibactam is a novel synthetic β-lactamase inhibitor with activity against Ambler class A [extended-spectrum β-lactamases (ESBLs), *Klebsiella pneumoniae* carbapenemases (KPCs)], class C (AmpC), and some class D [oxacillinases (OXA) β-lactamases]. However, CAZ/AVI is inactive against class B metallo-β-lactamases (MBLs), such as New Delhi metallo-β-lactamase (NDM), Imipenemase (IMP), and Verona integron-encoded metallo-β-lactamase (VIM) (Falcone and Paterson, 2016).

Although some studies have indicated that CAZ-AVI treatment for CR-GNB infections is more effective than polymyxins and tigecycline (van Duin et al., 2018; Fang et al., 2021; Shi et al., 2021), the risk factors associated with treatment failure of CAZ-AVI remain unclear, especially in this special population of KT recipients. Additionally, previous *in vitro* studies have demonstrated that CAZ-AVI, when used in combination with other drugs, exhibits high synergistic activity against CR-GNB (Gaibani et al., 2017; Wang et al., 2021; Zhang et al., 2024). However, it is unclear whether this combination is beneficial. Therefore, we conducted a retrospective study to assess the risk factors for clinical failure and death in CR-GNB-infected KT recipients treated with CAZ-AVI, as well as the effect of combination therapy.

## Materials and methods

### Study design and population

This single-center retrospective observational study was conducted at a 4990-bed tertiary care teaching hospital in Hefei, Anhui Province, China. From June 2019 to December 2023, clinical data from KT recipients (aged ≥ 18 years) with CR-GNB infection who received CAZ-AVI treatment for ≥ 72 h at the First Affiliated Hospital of Anhui Medical University were analyzed retrospectively. The primary outcome was 30-day mortality, with clinical and microbiological cures as secondary outcomes. The primary safety measure was the occurrence of treatment-related adverse events or resistance during or after CAZ-AVI treatment. All kidneys were donated voluntarily by relatives or deceased citizens with written informed consent in accordance with the Istanbul Declaration. The present study was approved by our institutional Ethics Review Committee and was conducted according to the principles of the Declaration of Helsinki.

### Data collection

The data were collected using the hospital’s electronic medical records system. The data collected included demographic characteristics (age, sex, and weight), coexisting conditions, infection-related characteristics (severity at onset of infection and source of infection), microbiological data, and treatment characteristics. Only the first course of CAZ-AVI treatment was considered if the recipient received more than one course. The Acute Physiology and Chronic Health Evaluation II (APACHE II) (LeGall et al., 1986) and Sequential Organ Failure Assessment (SOFA) were used to assess severity at the onset of infection (Vincent et al., 1996).

### Definition

The infection definition and classification standards used in the present study were those proposed by the Centers for Disease Control and Prevention (Horan et al., 2008). The date of infection onset was defined as the date of collection when the specimen became culture positive for the first time. The 30-day mortality was defined as all-cause mortality 30 days after the onset of infection. Clinical cure was defined as the resolution or significant improvement of the signs and symptoms of CR-GNB infection within 14 days of starting CAZ-AVI treatment. Clinical failure was defined as a failure to respond clinically and/or death. Microbiological cure was defined as the sterilization of site-specific cultures and/or blood cultures after treatment ended (the presence of a clinical cure in recipients without repeat samples was also considered a microbiological cure). Recurrence within 90 days of onset was defined as the occurrence of a second microbiologically confirmed CR-GNB infection in recipients whose initial infection was classified as clinically cured (with or without microbiologically confirmed). Combination therapy was defined as combining two or more antibiotics on a CAZ-AVI basis for at least 72 h, regardless of their *in vitro* activity. Salvage therapy was defined as treatment administered in the event of clinical failure of the first-line regimen or discontinuation of the first-line drug due to severe side effects (Tumbarello et al., 2019). Source control was defined as device removal or replacement in the event of urinary or vascular catheter infection or adequate drainage in the event of surgical site infection or abscess (Corbella et al., 2022).

### Immunosuppressive regimen and use of antibiotics

During the perioperative period, all recipients were given triple immunosuppressive therapy (tacrolimus or cyclosporine + prednisone + mycophenolate mofetil), and anti-thymocyte globulin (ATG) was added to some recipients for induction. The standard dose of CAZ-AVI was 2.5 g (ceftazidime 2000 mg and avibactam 500 mg), which was administered intravenously every 8 h for more than 2 h. Dosage and administration were adjusted according to kidney function according to the manufacturer’s recommendations (Rodríguez-Núñez et al., 2018).

### Microbiology

Susceptibility testing was performed using the VITEK-2 system (Biomerieux, Marcy-l’ Etoile, France) and the disc diffusion method. The minimum inhibitory concentrations (MICs) were interpreted according to breakpoints established by The Clinical and Laboratory Standards Institute (CLSI). According to CLSI criteria, carbapenem resistance was defined as resistance to ertapenem (MIC > 2 mg/ml) and resistance to imipenem or meropenem (MIC > 4 mg/ml) (Clinical and Laboratory Standards Institute, 2018).

### Statistical analysis

Statistical analysis was performed using SPSS software [Version 25.0; SPSS Inc., Chicago, IL, USA]. Continuous variables are presented as the mean ± standard deviation or median and interquartile range (IQR). Independent sample t tests and Mann–Whitney U tests were used to compare normally distributed and nonnormally distributed continuous variables, respectively. Categorical variables are presented as absolute frequencies (%) and were compared using the chi-square test or Fisher’s exact test if necessary. Variables with *P* values less than 0.1 in the univariable analysis were included in the multivariable analysis. Multivariate logistic regression analysis was used to identify independent risk factors for 30-day mortality and clinical cure. Hazard ratios (HRs) and 95% confidence intervals (CIs) were calculated for all associations. A *P* value < 0.05 was considered statistically significant. The Kaplan–Meier method was used for survival analysis.

## Results

According to the electronic medical records, 654 kidney transplants were performed in our center between June 2019 and December 2023, and 88 CR-GNB-infected recipients received CAZ-AVI treatment, of which four CR-GNB-infected recipients received CAZ-AVI for < 72 h and three CR-GNB-infected recipients aged < 18 years were excluded from the study. Finally, 81 recipients were included in the study.

### Baseline characteristics

The demographic and clinical characteristics of the recipients included in this study are shown in **Table 1**. The mean age of the recipients was 40.5 ± 7.7 years, 46.9% (38/81) were male, and the mean body mass index (BMI) was 21.9 ± 1.9 kg/m^2^. Sixty-seven (82.7%) recipients received organ donation after a citizen’s death. The etiologies of end-stage kidney disease included glomerulonephritis (n = 47, 58%), hypertension (n = 10, 12.3%), diabetes mellitus (n = 7, 8.6%), and others (n = 17, 21%). The median SOFA score at the onset of infection was 5 (interquartile range [IQR] 5.0-6.0), and the median APACHE II score was 10 (IQR 9.0-13.0).

**Table 1:** Characteristics of the 81 recipients receiving ceftazidime-avibactam treatment.

Sex, male, n (%)	38(46.9)
Age(years), mean±SD	40.5±7.7
BMI (kg/m2), mean±SD	21.9±1.9
Diabetes mellitus, n (%)	17(21)
Deceased donors, n (%)	67(82.7)
Etiology of kidney failure, n (%)	
HTA	10(12.3)
DM	7(8.6)
Glomerulonephritis	47(58)
Others	17(21)
Type of dialysis, n (%)	
hematodialysis	56(69.1)
peritoneal dialysis	25(30.9)
ATG induction, n (%)	53(65.4)
Types of infections, n (%)	
BSIs	35(43.2)
UTIs	29(35.8)
Pneumonia	32(39.5)
SSIs	36(44.4)
CRRT	41(50.6)
Source control, n (%)	20(24.7)
SOFA at infection onset, median (IQR)	5(5-6)
APACHIIat infection onset, median (IQR)	10(9-13)
combination therapy, n (%)	41(50.6)
CAZ-AVI as salvage therapy, n (%)	32(39.5)
Time from infection onset to CAZ-AVI initiation (days), median (IQR)	4(2-12)
Duration of CAZ-AVI treatment (days), median (IQR)	14(10-14)
CAZ-AVI initiation within 48h, n (%)	37(45.7)
Clinical cure, n (%)	59(72.8)
microbiological cure, n (%)	54(66.7)
30-day mortality, n (%)	18(22.2)
Recurrence within 90 days, n (%)	4(4.9)

BMI, body mass index; HTA, hypertension; DM, diabetes mellitus; ATG, anti-human thymocyte immunoglobulin; BSIs, bloodstream infections; UTIs, urinary tract infections; SSIs, surgical site infections; CRRT, continuous renal replacement treatment; SOFA, Sequential Organ Failure Assessment; APACHE II, Acute Physiologic Assessment and Chronic Health Evaluation II; CAZ-AVI, ceftazidime-avibactam.

### Infection characteristics and antimicrobial susceptibility

In a cohort of 81 CR-GNB-infected recipients, there were 61 cases of carbapenem-resistant *Klebsiella pneumoniae* (CRKP), 16 of CRPA, and 4 of CRAB infections. Surgical site infections (SSIs) (n = 36) were the most common source of infection, followed by bloodstream infections (BSIs) (n = 35), pneumonia (n = 32), and urinary tract infections (UTIs) (n = 29). Multiple site infections occurred in 40 recipients. **Table 2** presents the antimicrobial susceptibility results. All strains were resistant to meropenem and imipenem. Most strains were resistant to ceftazidime, levofloxacin, and gentamicin *in vitro*, with 0–18.7% sensitivity rates. In comparison, more strains demonstrated high sensitivity to amikacin (86.1%), polymyxin (100%), and tigecycline (100%) *in vitro*.

**Table 2:** Antimicrobial susceptibility of isolates from recipients with CR-GNB infections.

Antibiotic	Number of isolates tested(N)	Susceptible (%)
Ceftazidime	81	0.0
Levofloxacin	80	10.0
Gentamycin	80	18.7
Imipenem	81	0.0
Meropenem	81	0.0
Amikacin	79	86.1
Polymyxin	78	100.0
Tigecycline	81	100.0
Ceftazidime-Avibatam	81	100.0

CR-GNB, Carbapenem-resistant Gram-negative bacteria.

### Treatment characteristics

CAZ-AVI was given to 49 patients (60.5%) as first-line therapy, while it was given to 32 recipients (39.5%) as salvage therapy. CAZ-AVI was administered to 37 patients within 48 h of infection onset. Forty recipients (49.4%) received CAZ-AVI alone, 41 recipients (50.6%) received CAZ-AVI in combination with meropenem (n = 18, 43.9%), followed by imipenem-cilastatin (n = 12, 29.3%), tigecycline (n = 7, 17.1%), polymyxin (n= 3, 7.3%) and amikacin (n= 2, 2.4%). The median time from infection onset to CAZ-AVI administration was 4 days (IQR, 2–12), and the median duration of CAZ-AVI treatment was 14 days (IQR, 10–14). Appropriate source control was performed in 20 recipients (central venous catheter removal in 2 recipients with catheter-related BSIs, kidney allograft nephrostomy in 1 recipient with urinary tract obstruction, and percutaneous or surgical drainage in 17 recipients with SSIs).

### Outcomes

**Figure 1:**
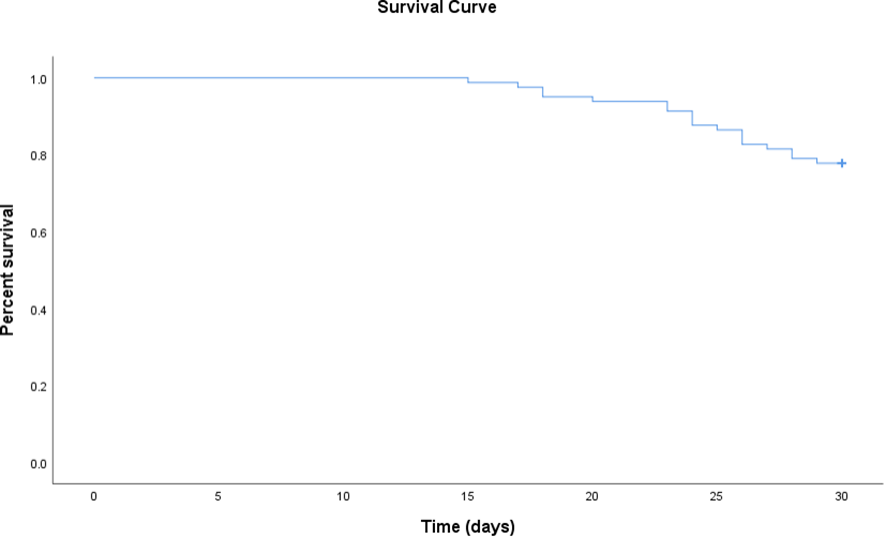
Survival curve of kidney transplant recipients treated with ceftazidime-avibactam for CR-GNB infections.

The 30-day mortality among the 81 CR-GNB-infected recipients treated with CAZ-AVI was 22.2% (18/81), and the survival curves of the 81 recipients are presented in **Figure 1**. The clinical cure rate was 72.8% (59/81), and the microbiological cure rate was 66.7% (54/81). In the multivariable analysis, the independent risk factor for 30-day mortality was a higher APACHE II score (OR: 4.517; 95% CI: 1.397-14.607; *P*=0.012) (**Table 3**). Clinical cure was positively associated with the administration of CAZ-AVI within 48 hours of infection onset (OR: 11.009; 95% CI: 1.344-90.197; *P* = 0.025) and negatively associated with higher APACHE II scores (OR: 0.700; 95% CI: 0.555-0.882; *P* = 0.002). (**Table 4**). No obvious differences were found in terms of clinical cure or 30-day mortality when CAZ/AVI was used as first-line or salvage therapy at the level of the whole cohort.

**Table 3:** Univariate and multivariate logistic regression analyses of risk factors associated with 30-day mortality.

Variable	Kidney transplant		Univariate analysis		Multivariate analysis	
	Death (N=18)	Survival (N=63)	OR (95%CI)	*P* value	OR (95%CI)	*P* value
Sex, male n (%)	6(33.3)	32(50.8)	0.484(0.162-1.451)	0.195		
Age (years), mean±SD	41.5±6.0	40.2±8.1	1.024(0.955-1.098)	0.512		
BMI (kg/m2), mean±SD	21.9±1.3	21.9±2.0	1.005(0.761-1.328)	0.971		
Diabetes mellitus, n (%)	5(27.8)	12(19.0)	1.635(0.488-5.471)	0.425		
Deceased donors, n (%)	12(66.7)	55(87.3)	0.291(0.085-0.994)	0.049	0.232(0.021-2.627)	0.238
Etiology of kidney failure, n (%)						
HTA	1(5.6)	9(14.3)	0.353(0.042-2.990)	0.339		
DM	1(5.6)	6(9.5)	0.559(0.063-4.969)	0.602		
Glomerulonephritis	13(72.2)	34(54.0)	2.218(0.706-6.963)	0.172		
Others	3(16.7)	14(22.2)	0.700(0.177-2.767)	0.611		
Type of dialysis, n (%)						
hematodialysis	14(77.8)	42(66.7)	1.750(0.512-5.978)	0.372		
peritoneal dialysis	4(22.2)	21(33.3)	0.571(0.167-1.952)	0.372		
ATG induction n (%)	10(55.6)	43(68.3)	0.581(0.199-1.696)	0.321		
Types of infections n (%)						
BSIs	11(61.1)	24(38.1)	2.554(0.871-7.485)	0.088	1.412(0.103-19.329)	0.796
UTIs	8(44.4)	21(33.3)	1.600(0.550-4.651)	0.388		
Pneumonia	11(61.1)	21(33.3)	3.143(1.064-9.280)	0.038	2.932(0.300-28.633)	0.355
SSIs	7(38.9)	29(46.0)	0.746(0.256-2.174)	0.591		
CRRT	9(50.0)	32(50.8)	0.969(0.340-2.762)	0.953		
SOFA at infection onset, median (IQR)	5(4.75-7.0)	5(5-6)	1.089(0.769-1.542)	0.630		
APACHIIat infection onset, median (IQR)	16.5(13-18.25)	10(9-12)	4.663(1.646-13.213)	0.004	4.517(1.397-14.607)	**0.012**
Source control, n (%)	4(22.2)	16(25.4)	0.839(0.241-2.922)	0.783		
combination therapy, n (%)	6(33.3)	35(55.6)	0.526(0.183-1.516)	0.234		
CAZ-AVI initiation within 48h, n (%)	5(27.8)	32(50.8)	0.373(0.119-1.169)	0.091	4.684(0.260-84.421)	0.295
Time from infection onset to CAZ-AVI initiation (days) median (IQR)	8(2-12.5)	4(2-12)	1.057(0.955-1.170)	0.286		
Duration of CAZ-AVI treatment (days) median (IQR)	14(12.25-14.25)	13(10-14)	1.087(0.944-1.250)	0.247		
CAZ-AVI as salvage therapy, n (%)	11(61.1)	21(33.3)	3.143(1.064-9.280)	0.038	3.658(0.122-109.963)	0.455

BMI, body mass index; HTA, hypertension; DM, diabetes mellitus; ATG, anti-human thymocyte immunoglobulin; BSIs, bloodstream infections; UTIs, urinary tract infections; SSIs, surgical site infections; CRRT, continuous renal replacement treatment; SOFA, Sequential Organ Failure Assessment; APACHE II, Acute Physiologic Assessment and Chronic Health Evaluation II. Bold indicates values <0.05.

**Table 4:** Univariate and multivariate logistic regression analyses of risk factors associated with clinical cure.

Variable	Kidney transplant		Univariate analysis		Multivariate analysis	
	Clinical cure (N=59)	Clinical failure (N=22)	OR (95%CI)	*P* value	OR (95%CI)	*P* value
Sex, male n (%)	29(49.2)	9(40.9)	1.396(0.518-3.763)	0.509		
Age (years), mean±SD	39.9±7.9	42.1±6.8	0.961(0.899-1.027)	0.243		
BMI (kg/m2), mean±SD	21.9±1.7	21.9±2.4	1.024(0.789-1.329)	0.857		
Diabetes mellitus, n (%)	11(18.6)	6(27.3)	0.611(0.195-1.919)	0.399		
Deceased donors, n (%)	51(86.4)	16(72.7)	2.391(0.721-7.923)	0.154		
Etiology of kidney failure, n (%)						
HTA	9(15.3)	1(4.5)	3.780(0.450-31.742)	0.221		
DM	4(6.8)	3(13.6)	0.461(0.094-2.248)	0.338		
Glomerulonephritis	34(57.6)	13(59.1)	0.942(0.348-2.545)	0.905		
Others	12(20.3)	5(22.7)	0.868(0.266-2.830)	0.814		
Type of dialysis, n (%)						
hematodialysis	40(67.8)	16(72.7)	0.789(0.267-2.338)	0.670		
peritoneal dialysis	19(32.2)	6(27.3)	1.267(0.428-3.751)	0.670		
ATG induction n (%)	40(67.8)	13(59.1)	1.457(0.531-4.003)	0.465		
Types of infections n (%)						
BSIs	24(40.7)	11(50.0)	0.686(0.256-1.834)	0.452		
UTIs	22(37.3)	7(31.8)	1.274(0.450-3.608)	0.648		
Pneumonia	21(35.6)	11(50.0)	0.553(0.205-1.489)	0.241		
SSIs	25(42.4)	11(50.0)	0.735(0.275-1.964)	0.540		
CRRT	30(50.8)	11(50.0)	1.034(0.389-2.754)	0.946		
SOFA at infection onset, mean±SD	5(5-6)	6(4.75-8)	0.732(0.524-1.002)	0.067	0.894(0.571-1.399)	0.624
APACHIIat infection onset, mean±SD	10(9-12)	13(11-17.25)	0.689(0.564-0.841)	<0.001	0.700(0.555-0.882)	**0.002**
Source control, n (%)	16(27.1)	4(18.2)	1.674(0.491-5.706)	0.410		
combination therapy, n (%)	33(55.9)	8(36.4)	2.221(0.810-6.094)	0.121		
CAZ-AVI initiation within 48h, n (%)	35(59.3)	2(9.1)	14.583(3.115-68.269)	0.001	11.009(1.344-90.197)	**0.025**
Time from infection onset to CAZ-AVI initiation (days) median (IQR)	2(2-12)	8(3.75-14)	0.908(0.825-1.000)	0.051	1.143 (0.923-1.416)	0.221
Duration of CAZ-AVI treatment (days) median (IQR)	14(10-14)	14(10.75-15)	0.963(0.846-1.096)	0.569		
CAZ-AVI as salvage therapy, n (%)	17(28.8)	15(68.2)	0.189(0.065-0.545)	0.002	0.317(0.035-2.849)	0.305

BMI, body mass index; HTA, hypertension; DM, diabetes mellitus; ATG, anti-human thymocyte immunoglobulin; BSIs, bloodstream infections; UTIs, urinary tract infections; SSIs, surgical site infections; CRRT, continuous renal replacement treatment; SOFA, Sequential Organ Failure Assessment; APACHE II, Acute Physiologic Assessment and Chronic Health Evaluation II. Bold indicates values <0.05.

Four recipients experienced recurrence of infection (assessed on day 90), with a recurrence rate of 4.9% (4/81). The CAZ-AVI MICs of the recurrent strains (three cases of CRKP and one case of CRPA) were not higher than those of the original strains. In addition, no strains developed CAZ-AVI resistance during the course of the study.

### Adverse effects

CAZ-AVI-related adverse events occurred in three recipients, including diarrhea (unrelated to *Clostridium difficile* infection) in one recipient while nausea and vomiting in two recipients, all of which recovered after symptomatic relief treatment, and no severe adverse reactions were observed.

## Discussion

This is the largest single-center study reported to date focused on KT recipients receiving CAZ-AVI for CR-GNB infection to explore the risk factors associated with clinical failure and 30-day mortality, as well as the effectiveness of combination therapy. The results showed that CAZ-AVI was administered to 81 CR-GNB-infected recipients, including 61 CRKP-infected recipients, 16 CRPA-infected recipients, and 4 CRAB-infected recipients. The 30-day mortality, clinical cure, and microbiological cure rates were 22.2%, 72.8%, and 66.7%, respectively, indicating that CAZ-AVI is effective in the treatment of CR-GNB infection after kidney transplantation.

The reported 30-day mortality of 22.2% in this study is consistent with the 30-day mortality reported in a recently published study involving 210 solid organ transplant recipients with bloodstream CRKP infection at 14 transplant centers in four countries, and they reported a 30-day mortality of 13.25% in recipients treated with CAZ-AVI (Pérez-Nadales et al., 2023). Another multicenter retrospective study from 2015 to 2019 at six medical centers in the United States included 203 CREs (n = 117) and CRPAs (n = 63) isolated from culture specimens, and the 30-day mortality in patients treated with CAZ-AVI was 17.2% (Jorgensen et al., 2019). However, Chen et al (Chen et al., 2021). reported 38.1% 30-day mortality in the liver transplant population, which was significantly higher than the 30-day mortality in our study (19.4%), which could be attributed to the difference in patient severity between studies. Our study revealed that 30-day mortality was associated with higher APACHE II scores, consistent with previous reports (Zhang et al., 2024). A meta-analysis conducted by Qian et al (Qian et al., 2021). reported that the average APACHE II score in nonsurviving patients was significantly higher than that in surviving patients at the time of diagnosis of CRKP infection, indicating that the APACHE II score is a critical and useful scoring system for clinicians to assess disease severity and predict the prognosis of patients with CR-GNB infection.

In addition, the clinical and microbiological cure rates in the present study were 72.8% and 66.7%, respectively, which were consistent with previous reports. Temkin et al (Temkin et al., 2017). reported a single-center case study in which 38 patients with CRE (89% CRKP) infection were treated with CAZ-AVI, with clinical and microbiological cure rates of 68.4% and 63.1%, respectively. In a multicenter study by King et al (King et al., 2017), which evaluated the clinical outcomes of 60 CRE (83% CRKP)-infected patients treated with CAZ-AVI, 65% and 53% achieved microbiological cure and clinical cure, respectively, at the end of CAZ-AVI treatment. The present study revealed that clinical failure was associated with higher APACHE II scores and failure to administer CAZ-AVI within 48 hours of infection onset, consistent with previous reports (Yu et al., 2023; Xu et al., 2024). A multicenter retrospective cohort study by Jorgensen et al (Jorgensen et al., 2019). demonstrated that CAZ-AVI initiation within 48 h of infection onset was associated with a lower clinical failure rate in patients with CR-GNB infection. Additionally, several studies have shown that the treatment of severe infections is time-sensitive, and delays in appropriate treatment have negative consequences (Seymour et al., 2017; Bonine et al., 2019), emphasizing the importance of rapid diagnostic testing in early pathogen identification and susceptibility testing. Continuous renal replacement therapy (CRRT) has previously been reported to be associated with clinical failure of CAZ-AVI treatment (Shields et al., 2018; Jorgensen et al., 2019); however, this association was not observed in our study, which may be attributed to our small sample size.

In the pre-CAZ-AVI era, combinations of two or more active antimicrobial agents were widely considered superior to single-agent regimens for treating CR-GNB, particularly for CRE infections associated with high mortality scores (Gutiérrez-Gutiérrez et al., 2017; Giannella et al., 2018). The role of CAZ-AVI as a combination therapy is still debated. A recent meta-analysis of 17 studies and 1435 patients (837 treated with CAZ-AVI combination therapy and 598 treated with CAZ-AVI monotherapy) treated with CAZ-AVI for CR-GNB infection revealed no significant difference in mortality, clinical cure rate, or microbiological cure rate between CAZ-AVI combination therapy and monotherapy, but monotherapy was more likely to present a trend toward posttreatment resistance than combination therapy (Li et al., 2021). Several studies have shown that CAZ-AVI combination therapy can reduce mortality compared with monotherapy, particularly in critically ill patients (Tumbarello et al., 2015; Zheng et al., 2021). In contrast, a small number of studies have reported the opposite results. Corbella et al (Corbella et al., 2022). reported an inverse correlation between combination therapy and clinical cure in 61 CRPA patients treated with CAZ-AVI. Karaiskos et al (Karaiskos et al., 2021). revealed that CAZ-AVI combination therapy was an independent risk factor for death in a multicenter prospective observational study, and this difference may be explained by the inclusion of patients with more severe infections in the combination group. In the present study, combination therapy showed no clear improvement in terms of 30-day mortality or clinical cure compared to monotherapy, consistent with the findings of most previous studies (Temkin et al., 2017; Li et al., 2021), indicating that CAZ-AVI is effective when administered alone. Compared with combination therapy, CAZ-AVI monotherapy may reduce the incidence of adverse events, particularly acute kidney injury, on the basis of the characteristics of combination therapy with aminoglycosides or colistin (Shields et al., 2016; King et al., 2017). No acute kidney injury events were observed in this study, which could be attributed to the fact that most CAZ-AVI combination antimicrobials used in the present study were carbapenems.

The recurrence rate within 90 days after onset in the present study was 4.9% (4/81), which was lower than the 11.7% to 21% reported in previous studies (Shields et al., 2016, 2018), and all four recurrent recipients were cured by CAZ-AVI retreatment. CAZ-AVI resistance was not detected in the present study, which is consistent with the findings of previous studies (Vena et al., 2020; Corbella et al., 2022). However, there is mounting evidence that resistance develops during CAZ-AVI exposure. Shields et al (Shields et al., 2016). reported that 10% (8/77) of CRE-infected patients developed resistance after receiving CAZ-AVI treatment for a median of 15 days (IQR, 7–31). Another study reported that 3.5% (20/577) of CRKP-infected patients had persistently positive cultures after CAZ-AVI initiation and eventually developed resistance to CAZ-AVI (Tumbarello et al., 2021). The most common cause of CAZ-AVI resistance is a point mutation in the *bla_KPC* gene, which can restore susceptibility to carbapenems, so some authors encourage clinicians to combine CAZ-AVI with carbapenems or other antibiotics to prevent resistance (Liu et al., 2022). The present study revealed CAZ-AVI to be safe, with an incidence of treatment-related adverse reactions similar to that previously reported (Balandín et al., 2022; Corbella et al., 2022), and no severe adverse drug reactions occurred.

This study has many limitations. First, because this was a single-center, retrospective study, the generalizability of our findings is limited. Second, since CAZ-AVI was introduced in China in 2019, our sample size was relatively small, which may have prevented us from identifying important risk factors for 30-day mortality and clinical failure, and larger randomized controlled trials are required to address this issue. Third, carbapenemase detection assays were not performed in this study due to the lack of necessary clinical laboratory equipment and reagents.

## Conclusion

This study identified the risk factors for clinical failure and 30-day mortality in CR-GNB-infected KT recipients treated with CAZ-AVI, and our study suggested that CAZ-AVI provides clinical benefit in terms of survival and clinical response in CR-GNB-infected KT recipients, even as a monotherapy. Higher APACHE II scores were associated with 30-day mortality and clinical failure, and timely administration of CAZ-AVI within 48 hours of infection onset was positively associated with a clinical cure. These findings may help clinicians optimize treatment strategies for KT recipients with CR-GNB infections using CAZ-AVI and reduce mortality in the future.

## Data availability statement

The datasets used and/or analysed during the current study are available from the corresponding author on reasonable request.

## Ethics statement

The studies involving humans were approved by the ethics committee of The First Affiliated Hospital of Anhui Medical University. The studies were conducted in accordance with the local legislation and institutional requirements. The ethics committee/institutional review board waived the requirement of written informed consent for participation from the participants or the participants’ legal guardians/next of kin because this study is a retrospective study. The animal study was approved by the ethics committee of The First Affiliated Hospital of Anhui Medical University. The study was conducted in accordance with the local legislation and institutional requirements.

## Author contributions

FZ: Conceptualization, Formal analysis, Investigation, Writing – original draft. PL: Conceptualization, Data curation, Formal analysis, Methodology, Writing – original draft. JZ: Conceptualization, Data curation, Formal analysis, Methodology, Writing – original draft. HD: Data curation, Formal analysis, Methodology, Software, Writing – original draft. GL: Conceptualization, Formal analysis, Writing – review & editing. CL: Conceptualization, Validation, Writing – review & editing.

## References

[B1] Balandín B. Ballesteros D. Pintado V. Soriano-Cuesta C. Cid-Tovar I. Sancho-González M.. (2022). Multicentre study of ceftazidime/avibactam for Gram-negative bacteria infections in critically ill patients. Int. J. Antimicrob. Agents 59, 106536. doi: 10.1016/j.ijantimicag.2022.106536 35091054

[B2] Bodro M. Sabé N. Tubau F. Lladó L. Baliellas C. González-Costello J.. (2015). Extensively drug-resistant *Pseudomonas aeruginosa* bacteremia in solid organ transplant recipients. Transplantation 99, 616–622. doi: 10.1097/TP.0000000000000366 25119130

[B3] Bonine N. G. Berger A. Altincatal A. Wang R. Bhagnani T. Gillard P.. (2019). Impact of delayed appropriate antibiotic therapy on patient outcomes by antibiotic resistance status from serious gram-negative bacterial infections. Am. J. Med. Sci. 357, 103–110. doi: 10.1016/j.amjms.2018.11.009 30665490

[B4] Chen F. Zhong H. Yang T. Shen C. Deng Y. Han L.. (2021). Ceftazidime-avibactam as salvage treatment for infections due to carbapenem-resistant *klebsiella pneumoniae* in liver transplantation recipients. Infect. Drug Resist. 14, 5603–5612. doi: 10.2147/IDR.S342163 34992387 PMC8710070

[B20] Clinical and Laboratory Standards Institute (2018). M100-S28. Performance standards for antimicrobial susceptibility testing: 28th informational supplement (Wayne, PA: Clinical and Laboratory Standards Institute).

[B5] Corbella L. Boán J. San-Juan R. Fernández-Ruiz M. Carretero O. Lora D.. (2022). Effectiveness of ceftazidime-avibactam for the treatment of infections due to *Pseudomonas aeruginosa* . Int. J. Antimicrob. Agents 59, 106517. doi: 10.1016/j.ijantimicag.2021.106517 34990760

[B6] Doi Y. (2019). Treatment options for carbapenem-resistant gram-negative bacterial infections. Clin. Infect. Dis. 69, S565–s575. doi: 10.1093/cid/ciz830 31724043 PMC6853760

[B7] Falcone M. Paterson D. (2016). Spotlight on ceftazidime/avibactam: a new option for MDR Gram-negative infections. J. Antimicrob. Chemother. 71, 2713–2722. doi: 10.1093/jac/dkw239 27432599

[B8] Fang J. Li H. Zhang M. Shi G. Liu M. Wang Y.. (2021). Efficacy of ceftazidime-avibactam versus polymyxin B and risk factors affecting clinical outcomes in patients with carbapenem-resistant *klebsiella pneumoniae* infections a retrospective study. Front. Pharmacol. 12. doi: 10.3389/fphar.2021.780940 PMC8703033 34955849

[B9] Gaibani P. Lewis R. E. Volpe S. L. Giannella M. Campoli C. Landini M. P.. (2017). *In vitro* interaction of ceftazidime-avibactam in combination with different antimicrobials against KPC-producing *Klebsiella pneumoniae* clinical isolates. Int. J. Infect. Dis. 65, 1–3. doi: 10.1016/j.ijid.2017.09.017 28951106

[B10] Giannella M. Trecarichi E. M. Giacobbe D. R. De Rosa F. G. Bassetti M. Bartoloni A.. (2018). Effect of combination therapy containing a high-dose carbapenem on mortality in patients with carbapenem-resistant *Klebsiella pneumoniae* bloodstream infection. Int. J. Antimicrob. Agents 51, 244–248. doi: 10.1016/j.ijantimicag.2017.08.019 28842283

[B11] Gutiérrez-Gutiérrez B. Salamanca E. de Cueto M. Hsueh P. R. Viale P. Paño-Pardo J. R.. (2017). Effect of appropriate combination therapy on mortality of patients with bloodstream infections due to carbapenemase-producing Enterobacteriaceae (INCREMENT): a retrospective cohort study. Lancet Infect. Dis. 17, 726–734. doi: 10.1016/S1473-3099(17)30228-1 28442293

[B12] Horan T. C. Andrus M. Dudeck M. A. (2008). CDC/NHSN surveillance definition of health care-associated infection and criteria for specific types of infections in the acute care setting. Am. J. Infect. Control 36, 309–332. doi: 10.1016/j.ajic.2008.03.002 18538699

[B13] Jorgensen S. C. J. Trinh T. D. Zasowski E. J. Lagnf A. M. Bhatia S. Melvin S. M.. (2019). Real-world experience with ceftazidime-avibactam for multidrug-resistant gram-negative bacterial infections. Open Forum Infect. Dis. 6, ofz522. doi: 10.1093/ofid/ofz522 31890725 PMC6934163

[B14] Karaiskos I. Daikos G. L. Gkoufa A. Adamis G. Stefos A. Symbardi S.. (2021). Ceftazidime/avibactam in the era of carbapenemase-producing *Klebsiella pneumoniae*: experience from a national registry study. J. Antimicrob. Chemother. 76, 775–783. doi: 10.1093/jac/dkaa503 33249436

[B15] King M. Heil E. Kuriakose S. Bias T. Huang V. El-Beyrouty C.. (2017). Multicenter study of outcomes with ceftazidime-avibactam in patients with carbapenem-resistant enterobacteriaceae infections. Antimicrob. Agents Chemother. 61, e00449-17. doi: 10.1128/AAC.00449-17 28483952 PMC5487633

[B16] LeGall J. R. Loirat P. Alpérovitch A. (1986). APACHE II—a severity of disease classification system. Crit. Care Med. 14, 754–755. doi: 10.1097/00003246-198608000-00027 3087704

[B17] Li D. Fei F. Yu H. Huang X. Long S. Zhou H.. (2021). Ceftazidime-avibactam therapy versus ceftazidime-avibactam-based combination therapy in patients with carbapenem-resistant gram-negative pathogens: A meta-analysis. Front. Pharmacol. 12. doi: 10.3389/fphar.2021.707499 PMC8476997 34594216

[B18] Liu C. Wu Y. Huang L. Zhang Y. Sun Q. Lu J.. (2022). The rapid emergence of ceftazidime-avibactam resistance mediated by KPC variants in carbapenem-resistant *klebsiella pneumoniae* in zhejiang province, China. Antibiotics (Basel) 11, 731. doi: 10.3390/antibiotics11060731 35740138 PMC9219983

[B19] Nang S. C. Azad M. A. K. Velkov T. Zhou Q. T. Li J. (2021). Rescuing the last-line polymyxins: achievements and challenges. Pharmacol. Rev. 73, 679–728. doi: 10.1124/pharmrev.120.000020 33627412 PMC7911091

[B21] Pérez-Nadales E. Fernández-Ruiz M. Natera A. M. Gutiérrez-Gutiérrez B. Mularoni A. Russelli G.. (2023). Efficacy of ceftazidime-avibactam in solid organ transplant recipients with bloodstream infections caused by carbapenemase-producing *Klebsiella pneumoniae* . Am. J. Transplant. 23, 1022–1034. doi: 10.1016/j.ajt.2023.03.011 37028515

[B22] Qian Y. Bi Y. Liu S. Li X. Dong S. Ju M. (2021). Predictors of mortality in patients with carbapenem-resistant *Klebsiella pneumoniae* infection: a meta-analysis and a systematic review. Ann. Palliat Med. 10, 7340–7350. doi: 10.21037/apm-21-338 34263631

[B23] Rodríguez-Núñez O. Ripa M. Morata L. de la Calle C. Cardozo C. Fehér C.. (2018). Evaluation of ceftazidime/avibactam for serious infections due to multidrug-resistant and extensively drug-resistant *Pseudomonas aeruginosa* . J. Glob Antimicrob. Resist. 15, 136–139. doi: 10.1016/j.jgar.2018.07.010 30036695

[B24] Seymour C. W. Gesten F. Prescott H. C. Friedrich M. E. Iwashyna T. J. Phillips G. S.. (2017). Time to treatment and mortality during mandated emergency care for sepsis. N Engl. J. Med. 376, 2235–2244. doi: 10.1056/NEJMoa1703058 28528569 PMC5538258

[B25] Shi Y. Hu J. Liu P. Wang T. Wang H. Liu Y.. (2021). Ceftazidime-avibactam-based versus tigecycline-based regimen for the treatment of carbapenem-resistant *klebsiella pneumoniae*-induced pneumonia in critically ill patients. Infect. Dis. Ther. 10, 2721–2734. doi: 10.1007/s40121-021-00542-3 34652713 PMC8517067

[B26] Shields R. K. Nguyen M. H. Chen L. Press E. G. Kreiswirth B. N. Clancy C. J. (2018). Pneumonia and renal replacement therapy are risk factors for ceftazidime-avibactam treatment failures and resistance among patients with carbapenem-resistant enterobacteriaceae infections. Antimicrob. Agents Chemother. 62, e02497-17. doi: 10.1128/AAC.02497-17 29507064 PMC5923134

[B27] Shields R. K. Potoski B. A. Haidar G. Hao B. Doi Y. Chen L.. (2016). Clinical outcomes, drug toxicity, and emergence of ceftazidime-avibactam resistance among patients treated for carbapenem-resistant enterobacteriaceae infections. Clin. Infect. Dis. 63, 1615–1618. doi: 10.1093/cid/ciw636 27624958 PMC5146720

[B28] Temkin E. Torre-Cisneros J. Beovic B. Benito N. Giannella M. Gilarranz R.. (2017). Ceftazidime-avibactam as salvage therapy for infections caused by carbapenem-resistant organisms. Antimicrob. Agents Chemother. 61, e01964-16. doi: 10.1128/AAC.01964-16 27895014 PMC5278727

[B29] Torres A. Zhong N. Pachl J. Timsit J. F. Kollef M. Chen Z.. (2018). Ceftazidime-avibactam versus meropenem in nosocomial pneumonia, including ventilator-associated pneumonia (REPROVE): a randomised, double-blind, phase 3 non-inferiority trial. Lancet Infect. Dis. 18, 285–295. doi: 10.1016/S1473-3099(17)30747-8 29254862

[B30] Tumbarello M. Raffaelli F. Giannella M. Mantengoli E. Mularoni A. Venditti M.. (2021). Ceftazidime-Avibactam Use for *Klebsiella pneumoniae* Carbapenemase-Producing K. pneumoniae Infections: A Retrospective Observational Multicenter Study. Clin. Infect. Dis. 73, 1664–1676. doi: 10.1093/cid/ciab176 33618353

[B31] Tumbarello M. Trecarichi E. M. Corona A. De Rosa F. G. Bassetti M. Mussini C.. (2019). Efficacy of Ceftazidime-Avibactam Salvage Therapy in Patients With Infections Caused by *Klebsiella pneumoniae* Carbapenemase-Producing K. pneumoniae. Clin. Infect. Dis. 68, 355–364. doi: 10.1093/cid/ciy492 29893802

[B32] Tumbarello M. Trecarichi E. M. De Rosa F. G. Giannella M. Giacobbe D. R. Bassetti M.. (2015). Infections caused by KPC-producing *Klebsiella pneumoniae*: differences in therapy and mortality in a multicentre study. J. Antimicrob. Chemother. 70, 2133–2143. doi: 10.1093/jac/dkv086 25900159

[B33] van Duin D. Bonomo R. A. (2016). Ceftazidime/avibactam and ceftolozane/tazobactam: second-generation β-lactam/β-lactamase inhibitor combinations. Clin. Infect. Dis. 63, 234–241. doi: 10.1093/cid/ciw243 27098166 PMC4928383

[B34] van Duin D. Lok J. J. Earley M. Cober E. Richter S. S. Perez F.. (2018). Colistin versus ceftazidime-avibactam in the treatment of infections due to carbapenem-resistant enterobacteriaceae. Clin. Infect. Dis. 66, 163–171. doi: 10.1093/cid/cix783 29020404 PMC5850032

[B35] Vena A. Giacobbe D. R. Castaldo N. Cattelan A. Mussini C. Luzzati R.. (2020). Clinical Experience with Ceftazidime-Avibactam for the Treatment of Infections due to Multidrug-Resistant Gram-Negative Bacteria Other than Carbapenem-Resistant Enterobacterales. Antibiotics (Basel) 9, 71. doi: 10.3390/antibiotics9020071 32050434 PMC7168189

[B36] Vincent J. L. Moreno R. Takala J. Willatts S. De Mendonça A. Bruining H.. (1996). The SOFA (Sepsis-related Organ Failure Assessment) score to describe organ dysfunction/failure. On behalf of the Working Group on Sepsis-Related Problems of the European Society of Intensive Care Medicine. Intensive Care Med. 22, 707–710. doi: 10.1007/BF01709751 8844239

[B37] Wang F. Zhou Q. Yang X. Bai Y. Cui J. (2021). Evaluation of ceftazidime/avibactam alone and in combination with amikacin, colistin and tigecycline against *Klebsiella pneumoniae* carbapenemase-producing *K. pneumoniae* by in *vitro* time-kill experiment. PloS One 16, e0258426. doi: 10.1371/journal.pone.0258426 34648556 PMC8516195

[B38] Xu C. Zeng F. Huang Y. Xu Q. Yang Y. Gong W.. (2024). Clinical efficacy of ceftazidime/avibactam combination therapy for severe hospital-acquired pulmonary infections caused by carbapenem-resistant and difficult-to-treat *Pseudomonas aeruginosa* . . Int. J. Antimicrob. Agents 63, 107021. doi: 10.1016/j.ijantimicag.2023.107021 37890733

[B39] Yahav D. Giske C. G. Gramatniece A. Abodakpi H. Tam V. H. Leibovici L. (2021). Erratum for Yahav et al., “New β-Lactam-β-Lactamase Inhibitor Combinations”. Clin. Microbiol. Rev. 34, e00021-21. doi: 10.1128/cmr.00021-21 33504504 PMC7849239

[B40] Yu J. Zuo W. Fan H. Wu J. Qiao L. Yang B.. (2023). Ceftazidime-avibactam for carbapenem-resistant gram-negative bacteria infections: A real-world experience in the ICU. Infect. Drug Resist. 16, 6209–6216. doi: 10.2147/IDR.S422545 37727274 PMC10506608

[B42] Zhang L. Ma Y. Zhao C. Zhao S. Zhao L. Yang Y.. (2024). Clinical outcomes and risk factors for death in critically ill patients with carbapenem-resistant *klebsiella pneumoniae* treated with ceftazidime-avibactam: A retrospective study. Infect. Drug Resist. 17, 239–248. doi: 10.2147/IDR.S445243 38293316 PMC10824611

[B41] Zhang F. Zhong J. Ding H. Pan J. Yang J. Lan T.. (2021). Analysis of risk factors for carbapenem-resistant *klebsiella pneumoniae* infection and its effect on the outcome of early infection after kidney transplantation. Front. Cell Infect. Microbiol. 11. doi: 10.3389/fcimb.2021.726282 PMC8535439 34692560

[B43] Zheng G. Zhang J. Wang B. Cai J. Wang L. Hou K.. (2021). Ceftazidime-avibactam in combination with *in vitro* nonsusceptible antimicrobials versus ceftazidime-avibactam in monotherapy in critically ill patients with carbapenem-resistant *klebsiella pneumoniae* infection: A retrospective cohort study. Infect. Dis. Ther. 10, 1699–1713. doi: 10.1007/s40121-021-00479-7 34241831 PMC8322179

